# Users of Internet Health Information: Differences by Health Status

**DOI:** 10.2196/jmir.4.2.e7

**Published:** 2002-11-22

**Authors:** Thomas K Houston, Jeroan J Allison

**Affiliations:** ^1^University of Alabama at BirminghamDivision of General Internal MedicineBirmingham ALUSA

**Keywords:** Internet, patient education, communication, health status

## Abstract

**Background:**

Millions of consumers have accessed health information online. However, little is known about their health status.

**Objective:**

To explore use of Internet health information among those who were sicker (fair/poor general health status) compared with those reported being healthier.

**Methods:**

A national, random-digit telephone survey by the Pew Internet & American Life Project identified 521 Internet users who go online for health care information. Our primary independent variable was general health status rated as excellent, good, fair, or poor. Patterns of Internet use, and types of information searched were assessed.

**Results:**

Among the 521 users, 64% were female, most (87%) were white, and median age was 42 years. Most individuals indicated that they learned something new online (81%) and indicated that they believe most information on the Internet (52%). Compared with those with excellent/good health, those with fair/poor health (N = 59) were relative newcomers to the Internet but tended to use the Internet more frequently, were more likely to use online chats, were less likely to search for someone other than themselves, and were more likely to talk about the new information with their physician (odds ratio 3.3 [95% confidence interval 1.8-6.3]), after adjustment for age, education and income.

**Conclusions:**

Health care professionals should be aware that their sicker patients are more likely to ask them about information they found online. Physicians, public health professionals, and eHealth developers should work together to educate patients about searching for health information online and to provide tools for them to navigate to the highest quality information.

## Introduction

Health information on the Internet is pervasive with thousands of Web sites, chat rooms, and support groups [1]. Some in the medical community have espoused the potential positive impact of the Internet on increasing health education and promoting self-care [2-4]. Others have cautioned about the public health risks of the varying quality of health information [5-8]. Despite these potential risks, millions of Americans have used the Internet to search for health information [9]. A previous survey using a convenience sample of primary care patients at one hospital-based practice suggests that most users rate the quality of Internet-based health information equivalent to information from their doctor [10]. Education of the public about how to evaluate the quality of the health information online is needed [11-13].

Users of Internet-based health information tend to reflect the higher-income, higher-education status associated with having Internet access [10]. However, little else is known about individuals who are searching for health information on the Internet. Are they mostly individuals with poor health and/or current illnesses (ie, our patients), or well individuals looking to stay well? Also, do the experiences online of patients with poor health differ from those without disease (eg, are sicker patients searching for different information, participating in support groups more often)? Answering these research questions may help physicians better understand what their patients are doing and may help public health practitioners better target their educational strategies about health information online.

To answer these questions, we took advantage of data collected in a national random-digit telephone survey by the Pew Internet & American Life Project related to use of health information online. Our objectives were to explore (1) Internet use characteristics of, (2) types of information sought by, and (3) impact of the Internet health information on the health care experience of individuals with poorer health (ie, fair/poor health) compared with those who reported better health.

## Methods

### Study Design

To obtain a representative sample of Americans who use the Internet, a national survey was conducted by the Pew Internet & American Life Project using a random-digit sample of telephone numbers selected from telephone exchanges in the continental United States. Between March and July 2000, Princeton Survey Research Associates conducted telephone interviews with Internet users 18 and over. Among these, 2027 individuals who used Internet-based health information were identified using the question, "Please tell me if you ever do any of the following when you go online - look for health or medical information?"

In August 2000, a follow-up telephone survey focusing on Internet health information use was conducted. After approximately 500 interviews were completed with individuals who had previously reported looking for health or medical information, recruitment was closed. While collecting this sample, an additional 144 individuals who participated in the original survey declined to participate. Data from the baseline and follow-up telephone interviews were stripped of unique identifying information for analysis. The Pew data are publicly available for download [14] and the authors received assistance in understanding the sampling frame and data structure from Susannah Fox at the Pew Internet & American Life Project and Jonathon Best of Princeton Survey Research Associates. The database included age, gender, race, education, income, a global rating of health status, patterns of Internet use, types of information searched and the impact of Internet health information on their knowledge and on their health care experience.

### Assessment of Health Status (Primary Independent Variable)

Our primary variable of interest, self-reported global health status, was based on a single question, "In general, how would you rate your own health — excellent, good, only fair, or poor?" Single global ratings of health status such as this have been recommended to reflect the wide variation in values of individuals and are in some respects superior to more complex measures [15,16]. A similar-format single-question global health rating is included on the SF-36, the National Health and Nutrition Examination Survey, and the Behavioral Risk Factor Surveillance System [17]. Single-item self-rated health status, or health-related quality of life, is as valid and reliable as more complex measurements and has been highly correlated with many diseases and health outcomes in previous studies [16,17]. Thus, individuals with global health ratings of fair or poor are likely to have chronic disease or acute medical illnesses and higher mortality [17-19].

### Patterns of Internet Use, Type of Health information, and Impact

To assess patterns of Internet health information use, participants were asked when they started using the Internet, how frequently they used the Internet to look for advice or information about health or health care, and the number of Web sites they visited the last time they went looking for health information. Participants were also asked if they believe the information they see on the Internet, if they participated in online chat rooms and whether they were looking for health information online for themselves or someone else. The type of information searched, such as general health information, information about fitness or nutrition, or specific information on a health condition, doctor, or hospital was assessed. The impact of Internet health information on knowledge was assessed by asking if participants had learned anything new from the online health information. Participants were then asked if the health information "has improved the way you take care of your health." Because Internet health information may have an impact on the physician-patient relationship, the survey also included the question, "Did you later talk to a doctor or nurse about the information you got online?"

### Analysis

First, demographic characteristics including age, gender, race, income, and education were compared among those with health status ratings of excellent, good, and fair/poor using Mantel-Haenzel χ^2^ trend statistics. To compare our sample of Internet users with other patient surveys, the percentages of individuals with fair or poor health status and associated demographic characteristics in this sample were compared with the percentages noted in the year 2000 Behavioral Risk Factor Surveillance System (BRFSS) [20].

The patterns of demographic characteristics associated with global health status in our study were used to confirm the reliability of this measure compared with previous studies. Health status rating has been associated with education, age, and income in previous research [17,21,22].

The frequency of those with fair/poor, good, and excellent health status reporting each pattern, type of information, and impact variable described above was compared using Mantel-Haenzel χ^2^ trend statistics [23]. Responses to questions related to pattern of Internet use were dichotomized based on distribution of responses for use as dependent variables in logistic regression. For variables associated with health status in univariate analysis at *P*<= .2, a series of logistic regression analyses were used to assess the association of our primary independent variable, health status, with each of the dichotomized pattern, type of information and impact dependent variables after adjustment for demographic characteristics. Each model was developed by introducing variables individually and then in combination to assess for evidence of interaction. To test for significance of trend across health status categories, health status was incorporated into the models as a continuous variable. The Pearson χ^2^ statistic was calculated for each multivariable model to test goodness of fit, and area under the Receiver Operating Characteristics curve (c statistic) was also calculated to assess discriminative power [23,24]. Pearson χ^2^ *P*> .1 indicates an adequate fit of the model to the data.

## Results

Our sample of 521 Internet users who access health information online identified from this national survey were mostly female (N = 331 [64%]) and had a median age of 42 years. Only 38 individuals (7%) were African American, 5 were Asian, and 20 were other nonwhite races. Compared with the original sample of 2027 Internet health information users, the 521 individuals who agreed to the follow-up survey were similar in ethnic distribution, educational level, and their frequency of Internet use, but those who completed the follow-up were slightly older (median age 42 vs 39, *P*< .01).

Ninety-nine percent (N = 520) of the participants in the focused Internet health information follow-up survey rated their health status. Based on this single-item global health status question, we identified 59 individuals (12%) with fair/poor health, 257 (49%) with good health, and 204 (39%) with excellent health. Associations of health status with demographics are summarized in Table 1. Compared to the 12% with fair/poor health in this sample, a similar 13.5% of the respondents to the 2000 BRFSS, reported fair or poor health. However, only 28% of individuals in the BRFSS were college graduates compared with 46% in our sample; and 33% of BRFSS participants had household incomes over $50,000 compared with 48% in our study.

**Table 1:** Demographic characteristics by health status among Internet health information users*

		Health Status		
	N* (%)	Excellent (%)	Good (%)	Fair/Poor (%)
Overall	521	204 (39)	257 (49)	59 (12)

Gender				
Male	189 (36)	69 (34)	95 (37)	25 (42)
Female	331 (64)	135 (66)	162 (63)	34 (58)
Race				
White	437 (87)	171 (88)	216 (86)	50 (88)
Black	38 (8)	13 (7)	20 (8)	5 (9)
Other	25 (5)	9 (5)	14 (6)	2 (5)
Age				
18-34	149 (29)	60 (29)	177 (30)	12 (20)
35-54	297 (54)	114 (56)	150 (58)	33 (56)
55 and older	74 (14)	30 (15)	30 (12)	14 (23)
Education†				
Less than college	272 (54)	93 (47)	136 (54)	43 (75)
College graduate	233 (46)	103 (53)	116 (46)	14 (25)
Income‡				
Less than $30,000	154 (30)	54 (26)	73 (28)	26 (44)
$30,000-$50,000	117 (22)	39 (19)	65 (25)	13 (22)
Over $50,000	250 (48)	111 (54)	119 (46)	20 (34)
Married				
Yes	343 (32)	139 (29)	167 (34)	37 (35)
No	161 (68)	57 (71)	84 (66)	20 (65)

^*^ Total N varies slightly due to small number of missing values (less than 2%).

^†^ Mantel-Haenzel χ^2^ test for trend *P*< .01.

^‡^ Mantel-Haenzel χ^2^ test for trend *P*< .05.

### Health Status and Patterns of Internet Use

**Figure 1:**
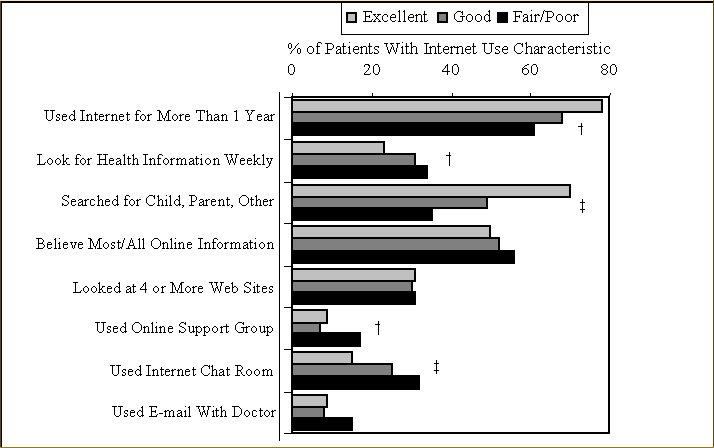
Health status and Internet use characteristics among Internet health information users

A significant dose-response association was seen with shorter history of Internet use and lower health status (Figure 1). Compared with those in excellent health, those with fair/poor health and those with good health were less likely to have begun using the Internet over a year ago after adjusting for education, age, and income in multivariable logistic regression — see OR (odds ratio) and CI (Confidence Interval) data in Table 2: OR is 0.5 (95% CI, 0.2-1.00) and 0.6 (95% CI, 0.4-0.9) for fair/poor health and good health respectively; *P* for trend < .01.

In contrast, there was a stepwise trend toward more-frequent current use of the Internet for health information among those with poorer health. Those with fair/poor health status were more likely to participate in online chat rooms compared with those with excellent health, but were less likely to have looked for health information for someone other than themselves (Figure 1,Table 2).

**Table 2:** Multivariable analyses of health status and Internet use patterns, types of information sought, and impact on health care communication among Internet health information users*

			Goodness of fit	
	Health Status	Adjusted Odds ratio†	Pearson's χ^2^	c
Used the Internet over a year ago‡	Excellent	Referent		
	Good	0.6 (0.4-0.9)		
	Fair/Poor	0.5 (0.2-1.00)	0.2	0.6
Use the Internet to look for health information about once a week§	Excellent	Referent		
	Good	1.54 (1.01-2.37)		
	Fair/Poor	1.77 (0.91-3.41)	0.2	0.6
Searched for health information for a child, parent or someone else‡	Excellent	Referent		
	Good	0.3 (0.3-0.6)		
	Fair/Poor	0.2 (0.1-0.4)	0.7	0.7
Participated in online support group for people who are concerned about the same health issues	Excellent	Referent		
	Good	0.7(0.4-1.4)		
	Fair/Poor	2.3(1.0-5.6)	0.7	0.6
Used online chat rooms‡	Excellent	Referent		
	Good	1.82 (1.1-2.0)		
	Fair/Poor	2.7 (1.3-5.5)	0.3	0.7
Sought information about specific doctors, or hospitals‡	Excellent	Referent		
	Good	2.2 (1.1-4.8)		
	Fair/Poor	3.2 (1.2-8.9)	0.4	0.6
Sought information about medicines or treatments for an illness or conditionSECT	Excellent	Referent		
	Good	1.5 (0.9-2.4)		
	Fair/Poor	2.2 (1.1-4.5)	0.3	0.6
Discussed Internet information with Physician or Nurse?‡	Excellent	Referent		
	Good	1.2 (0.8-1.8)		
	Fair/Poor	3.3 (1.8-6.3)	0.2	0.6

^*^ Total N varies slightly due to small number of missing values (less than 2%)

^†^ Logistic regression models developed for each Internet information "pattern/type/impact" characteristic associated with health status at *P*<= 0.2 in univariate χ^2^. Each adjusted odds ratio is from a separate logistic regression analysis with dichotomized Internet information "pattern/type/impact" characteristic as dependent variable and with health status as the primary independent variable, adjusted for income, age, education.

^‡^ Test for trend *P*< .01.

^§^ Test for trend *P*< .05.

The majority (52%) of these 521 Internet health information users indicated that they could believe most of the information on the Internet and this did not differ by health status. Only 30% had visited more than 4 Web sites the last time they searched for health information and this did not differ by health status. Few individuals (N = 49 [9%]) were using e-mail with their doctors (Figure 1).

### Type of Information Searched

**Figure 2:**
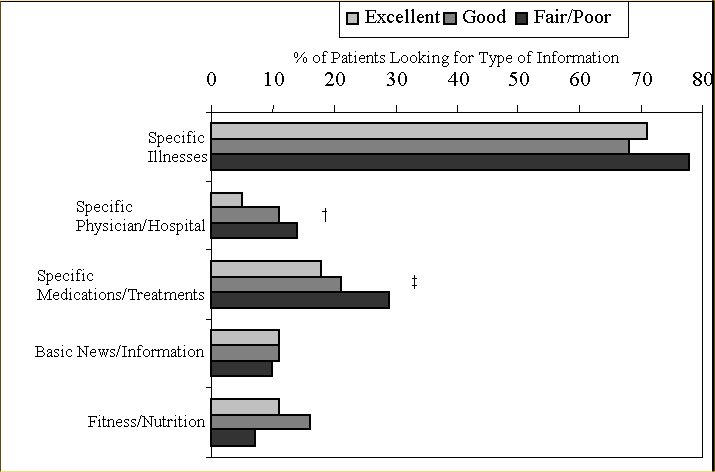
Health status and type of information searched among Internet health information users

Participants were asked to describe the information they were looking for the last time they went online for health information (Figure 2). A consistent, stepwise association of lower health status with more frequent reporting of searching for information about specific physicians, hospitals, medications, and treatments was seen. All groups frequently reported looking for information about specific illnesses. In multivariable analysis, those with poorer health status were again more likely to be searching for specific health information the last time they went online (Table 2).

### Impact of Internet Health Information

**Figure 3:**
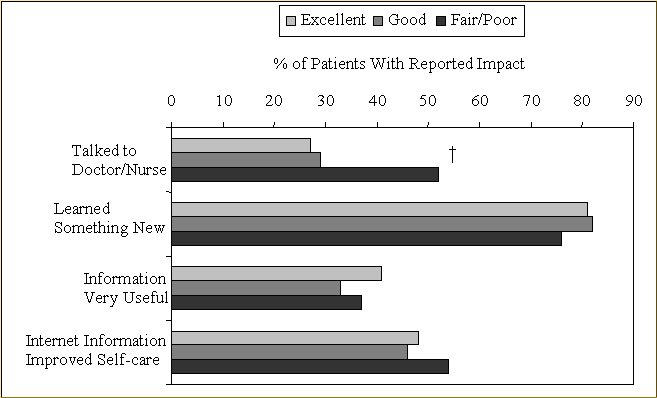
Health status and reported impact of online health information among 520 Internet health information users

Most individuals (N = 420 [81%]) indicated that they "learned something new" the last time they went online (Figure 3). This report of increased knowledge did not seem to vary by health status. Health status was not related to the self-reported usefulness of the Internet health information. However, the majority (52%) of the 59 individuals with fair/poor health status reported later talk to a doctor or nurse about the Internet health information, whereas less than a third of those with higher health status reported talking to a doctor or nurse. After adjustment for age, gender, and education, those with fair/poor health were considerably more likely to communicate with a health care provider (OR 3.3 [95% CI, 1.8-6.3]) compared with those with excellent health (Table 2).

## Discussion

In this telephone survey of 520 current Internet health information users, the majority of individuals reported that they learned something new and trusted the information they found. Consistent with our results, prior surveys of primary care patients using the Internet for health information also suggested that most users rate the quality of information as very good or excellent [10]. Our analysis also provides new data that significant differences exist between sicker patients and those with better self-reported health status in that sicker patients were more-frequent users of Internet health information, more likely to search for specific information, more frequently participating in chats, and more likely to discuss the information they found online with their health care provider.

Main Findings

Over half (52%) of the individuals reported that they could believe all or almost all the information online, but a minority (30%) reported "comparison shopping" for information by looking at multiple Web sites to gather information the last time they went foraging for health information. This provides further evidence that additional public health strategies should be developed to teach users about the variation in quality of information and to help them find quality online information. The majority (80%) of our 520 health information seekers found the information through a search engine. The effectiveness of searching through a search engine is limited, with only 20% of the top links leading to relevant content [8]. Although quality information does exist on the Internet, one systematic review indicated that 24% of the clinical elements felt important by experts were not included in the Web sites found by major search engines [8]. Some Web sites have begun to voluntarily comply with standards of ethics and quality [25]. Current research aims to develop digital quality seals that can be assigned by third-party raters and help consumers navigate to the best information [26,27]. Efforts to evaluate Web sites and accredit those who meet standards are also ongoing [28,29].

Those with fair/poor health were more likely to search for specific information on their doctor and medications and were more likely to speak to their health care provider about the information they found online. Providers should anticipate that their patients with chronic illnesses may present with information from the Internet. Because the sicker patients were relative newcomers to the Internet and currently frequently used the Internet to find health information, they may be particularly at risk for accessing less than optimal-quality health information. Physicians are a particularly valued source of information for patients and thus the office visit may be an excellent opportunity to educate patients about the variable quality of health information available and to direct patients toward higher-quality information. Thus, physicians should also be educated about Internet-based health information so they may better teach their patients.

The global health status assessment used in this study was reliable when compared to previous studies. The pattern of demographics associated with lower health status, suggests that the health status variable is functioning as seen in previous studies [21,22]. Also, the percentage of individuals with fair or poor health in this study was similar to that seen in the Behavioral Risk Factor Surveillance System [20]. Participants in our sample did vary from those in the BRFSS in that they were more educated and from a higher socio-economic status. .It is possible that other chronically ill patients with lower socio-economic status would be motivated to search for health information online but do not have access to the information due to the disparities in Internet access.

The demographics of many sicker patients (ie, lower income and lower education) identified in this and other research may make those with chronic disease particularly vulnerable to the disparities in access and barriers to understanding the various health Web sites [30]. In addition to the limits of access due to the "digital divide," health literacy also limits access to online health information [8,30]. Further research is needed to extend Internet access to those on the wrong side of the digital divide and to expand the range of Web sites for those with lower health literacy [31-33].

Strengths and Limitations

Our study has several limitations. The survey did not record specific diseases. Although consistent with prior studies, it is possible that using the measure of health status as a surrogate for illness and chronic disease has resulted in some misclassification [21,22]. In addition, the exact Web sites, chat rooms, and search engines that individuals were visiting were unknown. This study is a cross-sectional assessment and inferences of causality cannot be made. Our project focused on current Internet information seekers and is thus not generalizable to individuals who are not currently using the Internet for health information.

A strength of this study is the random-digit, population-based method used to identify this group of users. This increases the likelihood that our sample is representative of the population of Internet users, and thus enhances generalizability. Although this method of sampling misses individuals without telephones, we think it unlikely that many households without telephones have Internet access. Based on a search of the National Library of Medicine's PubMed database as of June 2002, the current study is the first to assess the particular patterns, type of information, and impact of online health information on those with poor health status. Previous research on use of the Internet among patients has been based on convenience samples [10]. In addition, the level of detail within this exploratory analysis provides pilot data on which to build future research related to tailoring information to the health information needs of those with poor health status.

Conclusion

Our study provides preliminary data on the experiences of online health information seekers. Although the majority of participants were in good health, those individuals with apparent illnesses were more-frequent users of the health information, and were more likely to combine their information seeking with their health care experience. Because the sicker patients are frequent users of specific Internet health information, they may be a population especially vulnerable to the varying availability and quality of Internet health information.

Very few individuals had used the Internet as a portal to communicate with their health care providers, but those with fair/poor health were more likely to communicate in person with their health care providers about the Internet health information they found. Thus, health care professionals should be aware that their patients with lower health status who have used the Internet for health information are likely the ones to come to them to discuss the information they have found. When presented with health information from the Internet, physicians can use this as a "teachable moment" and take the opportunity to educate their patients about the variability of information quality, and point patients toward appropriate sites. Physicians, public health professionals, and eHealth developers should work together to educate patients about searching for health information online and to provide tools for them to navigate to the highest quality information. Future studies should prospectively assess the impact of Internet-based health information on health care utilization and outcomes.

## Abbreviations

BRFSS: Behavioral Risk Factor Surveillance System
OR: Odds Ratio
